# Residential green space improves cognitive performances in primary schoolchildren independent of traffic-related air pollution exposure

**DOI:** 10.1186/s12940-023-00982-z

**Published:** 2023-03-30

**Authors:** Nelly D. Saenen, Tim S. Nawrot, Pauline Hautekiet, Congrong Wang, Harry A. Roels, Payam Dadvand, Michelle Plusquin, Esmée M. Bijnens

**Affiliations:** 1 Centre for Environmental Sciences, Hasselt University, Hasselt, Belgium; 2 Department of Public Health and Primary Care, Leuven University, Louvain, Belgium; 3 Risk and Health Impact Assessment, Sciensano (Belgian Institute of Health), Brussels, Belgium; 4 Louvain Centre for Toxicology and Applied Pharmacology, Université Catholique de Louvain, Brussels, Belgium; 5 ISGlobal, Barcelona, Spain; 6 Pompeu Fabra University, Barcelona, Catalonia Spain; 7 Ciber On Epidemiology and Public Health (CIBERESP), Madrid, Spain; 8 Department of Human Structure and Repair, Ghent University Hospital, Ghent, Belgium; 9 Department of Environmental Sciences, Faculty of Science, Open University, Heerlen, The Netherlands

**Keywords:** Neurodevelopment, Greenspace, Brain development, Natural environment, Mental health

## Abstract

**Background:**

Cognitive performances of schoolchildren have been adversely associated with both recent and chronic exposure to ambient air pollution at the residence. In addition, growing evidence indicates that exposure to green space is associated with a wide range of health benefits. Therefore, we aimed to investigate if surrounding green space at the residence improves cognitive performance of primary schoolchildren while taking into account air pollution exposure.

**Methods:**

Cognitive performance tests were administered repeatedly to a total of 307 primary schoolchildren aged 9-12y, living in Flanders, Belgium (2012–2014). These tests covered three cognitive domains: attention (Stroop and Continuous Performance Tests), short-term memory (Digit Span Forward and Backward Tests), and visual information processing speed (Digit-Symbol and Pattern Comparison Tests). Green space exposure was estimated within several radii around their current residence (50 m to 2000 m), using a aerial photo-derived high-resolution (1 m^2^) land cover map. Furthermore, air pollution exposure to PM_2.5_ and NO_2_ during the year before examination was modelled for the child’s residence using a spatial–temporal interpolation method.

**Results:**

An improvement of the children’s attention was found with more residential green space exposure independent of traffic-related air pollution. For an interquartile range increment (21%) of green space within 100 m of the residence, a significantly lower mean reaction time was observed independent of NO_2_ for both the sustained-selective (-9.74 ms, 95% CI: -16.6 to -2.9 ms, *p* = 0.006) and the selective attention outcomes (-65.90 ms, 95% CI: -117.0 to -14.8 ms, *p* = 0.01). Moreover, green space exposure within a large radius (2000 m) around the residence was significantly associated with a better performance in short-term memory (Digit-Span Forward Test) and a higher visual information processing speed (Pattern Comparison Test), taking into account traffic-related exposure. However, all associations were attenuated after taking into account long-term residential PM_2.5_ exposure.

**Conclusions:**

Our panel study showed that exposure to residential surrounding green space was associated with better cognitive performances at 9–12 years of age, taking into account traffic-related air pollution exposure. These findings support the necessity to build attractive green spaces in the residential environment to promote healthy cognitive development in children.

**Supplementary Information:**

The online version contains supplementary material available at 10.1186/s12940-023-00982-z.

## Introduction

Human contact with green spaces may play a crucial role in brain development that can lay the foundation for an improved mental condition and a healthy life [1, 2]. Interacting with nature has already been linked to a myriad of beneficial effects troughout life: lower risk of adverse birth outcomes [3], enhanced cognitive development in children [4], improved mental health [5], decreased risk of chrionic diseases (e.g. cardiovascular conditions and diabetes) [6], improved healthy ageing [7], and reduced mortality [8].

In recent years, also an improvement has been observed in mental health [9, 10], and emotional status [11]. In addition, exposure to surrounding green space at school has been associated with better school performance in children, but supporting evidence was mostly based on an ecological study with a school-level research design [12]. An increasing number of studies have investigated the association of cognitive development at the individual level with residential green spaces during childhood. One study from the UK found that a higher quality of neighbourhood green space was related to a better spatial working memory among 4,758 eleven-year-old children living in urban areas [13]. Among 456 Belgian children aged 4 to 6 years old, increased residential green space was associated with improved attention, psychomotor speed and visual recognition/working memory [14]. Two studies conducted in Spain observed better attention test scores in association with higher lifelong residential greenness [15] and enhanced progress in working memory and attention associated with higher total greenness including school, commuting route, and residence [16]. A longitudinal study among Italian children at the age of 7 years found progress in attention, concentration, and numerical reasoning for an increase in residential surrounding greenness within 500 m [17]. The observed association was mediated in part by a reduction in NO_2_. Contrary to the findings mentioned above, residential greenness exposure during childhood was not associated with attention, working memory and cognitive function within a multi-centric birth cohort study spread over 6 European countries [18]. Further evidence is provided by epidemiological research showing a positive association between residential green space and the intelligence of children [19, 20]. An overview of studies investigating greenness in association with cognitive development in children can be found in the systematic reviews by Luque-García et al. and by Buczylowskad et al*.* 2023 [21, 22].

According to Markevych. et al*.* (2017), the potential beneficial effects of green spaces on health can be classified into three domains that emphasize the general functions of green space: restoring capacities (attention restoration and psychophysiological stress recovery), building capacities (promoting physical activity and facilitating social interaction and cohesion), and reducing harm (reducing exposure to environmental stressors such as air pollution, noise, and heat) [23]. Together, all these factors can positively impact cognitive performance. On the other hand, exposure to air pollution has been associated with impaired cognitive performance among children [24–26]. Considering the interrelation between air pollution and greenness, the capacity of green vegetation to directly remove pollutants from the air by deposition is considered small [27]. However, green barriers might reduce the dispersion of air pollution. Another, explanation for the negative association between green space and air pollution is that there are fewer sources of air pollution within greener areas [23].

One of the main challenges as to the impact of green space on children’s cognitive development is to unravel the independent role of green space exposure from other confounding variables [28]. In the COGNAC (COGNition and Air pollution in Children) panel study on primary schoolchildren in Flanders (Belgium), we previously found that differential cognitive performances were consistently and adversely associated with recent and chronic ambient exposure to air pollution at the children’s residence [29]. Within this current study, we hypothesized that the surrounding green space at the residence of the same population of primary schoolchildren improves the cognitive performance. Finally, we evaluated this association while taking into account residential exposure to air pollution indicators one year before examination.

## Materials and methods

### Study population

This study was conducted in the framework of the COGNAC study, in which children aged 9–12 years were enrolled from three primary schools in three different study areas (Tienen, Zonhoven, Hasselt) in Flanders, Belgium [29]. Two schools (Zonhoven, Hasselt) are located within a suburban region close to a city and the third school (Tienen) is located in an urban area. In total, 770 children were invited of which 334 children participated (43%) in the study between January 2012 and February 2014. The parents of the participants were asked to fill out a questionnaire to collect additional information about the previous and current residential address, the exposure to passive tobacco smoke, the maternal education, the socioeconomic status of the family, and the child’s ethnicity. The socioeconomic status was assessed by using the mother’s education (no or primary education, secondary education, or college or university degree) and the highest rank of occupation of either parent (unqualified worker or unemployed; qualified worker, white-collar assistant, or teaching staff; self-employed, specialist, or member of management). Information on neighbourhood socioeconomic status (continuous) was defined based on annual household income in 2012 within the statatistical sector of residence and derived from Belgian census data (FOD Economie/DG Statistiek). Country of birth of the parents (both parents born in Belgium, one or both parents born abroad) was used as an indicator of ethnicity. Passive smoking was defined as exposure to indoor tobacco smoke when one or more family member(s) smoked inside the house. The height and weight of the children were recorded and body mass index (BMI) was calculated.

From the 334 children who agreed to participate in this study, we had to exclude 27 children with missing data on cognitive tests, mother’s education and/or occupation of the parents, BMI, passive smoking exposure, or residential address. For the statistical analysis, 307 children were included: 276 children (89.9%) were examined at three-time points, 28 children (9.1%) at two-time points, and 3 children (1.0%) at one-time point, aggregating to a total of 887 examinations. Written informed consent was obtained from the parents and oral consent was given by the children.

### Cognitive performance tests

Within this panel study, the schoolchildren repeated the cognitive performance tests on three occasions to deal with the learning effect. Cognitive performance was evaluated by administering in the following order a computer version of the Stroop Test [30] and four tests from the Neurobehavioral Evaluation System 3 (NES3) Battery: Continuous Performance, Digit Span, Digit-Symbol, and Pattern Comparison [31, 32]. The cognitive assessment lasted about 20 min. The order of the test was exactly the same on each occasion. The examinations took place on Monday, Tuesday, and Friday between 9:00 a.m. and 2:00 p.m. The mean (SD) time interval between two consecutive examinations was 41 (22) days. The interviewer had an appropriate training for administrating the cognitive tests. The Neurobehavioral Evaluation System 3 (NES3) Battery and Stroop Test use digitally recorded speech instructions to verbally guide the participants. This eliminates observer bias and the need of highly-trained interviewers, as is the case for manual-testing [31]. The room where the examinations took place was quiet, appropriately lighted, and ventilated. The outside temperature on the day of the examination was on average (± SD) 2.9° C (± 5.1) and ranged from − 6.4 to 9.1° C.

- Sustained and selective attention was tested with the Continuous Performance Test whereby silhouettes of animals (e.g. cat) are shown on the screen, sequentially for 200 ms. The task consists of responding immediately by pressing the spacebar if the cat’s silhouette is shown but not the silhouette of a different animal. A new animal silhouette is shown after 1000 ms. Besides mean reaction time, the continuous performance test also provides information on the standard deviation of the reaction time across blocks as a measure of consistency in responding and ability to sustain attention over time. However, in this study we used the mean reaction time as this is a measure frequently reported [33] and easier to interpret.

- To test the selective attention of the children we used the Stroop Test. During the Stroop Test, a total of four buttons are displayed on the screen (red, blue, yellow, and green) and the name of these colours appears on the screen in a different colour than the name indicates. As fast as possible the children needed to touch the button that has the same colour as the name indicates, ignoring the colour of the printed name. Before the beginning of the test, a total of eight practice trials were taken followed by 48 test trials. The mean reaction time was only calculated if the total number of test trials with wrong responses was smaller than or equal to 16.

- Testing of the short-term memory with the Digit Span Test included two parts. The first part is to reproduce a sequence of digits after an audible presentation in the order of the presented digits. In the beginning, three digits are given and in case of a correct answer, a one-digit longer sequence is presented. The test will continue until two successive incorrect answers are given. The second part of the test consists of reproducing the digits in the reverse order of the presentation.

- The visual information processing speed was tested with the Digit-Symbol Test and the Pattern Comparison Test. In the Digit-Symbol Test, a row of nine symbols together with nine digits is displayed at the top of the screen. During the test, 27 digits will appear in succession on the screen. When a digit is displayed, the task is to pinpoint as fast as possible which symbol pairs with the displayed digit in the row of symbols at the base of the screen. A new digit appears only after the correct symbol has been pinpointed. In the subsequent test, the Pattern Comparison Test, three matrices are displayed consisting of 10 × 10 blocks. Two of the three matrices are identical and the task is to indicate the pattern that is different from the other two patterns. In total, the test includes 25 items.

Each test outcome was characterized by a performance parameter, including the mean reaction time (milliseconds, msec) for the Continuous Performance Test and the Stroop Test, the maximum forward and backward span (number of digits) for the Digit Span Test, the total latency (seconds, sec) for the Digit-Symbol Test, and the average latency (sec) for the Pattern Comparison Test. A lower mean reaction time indicates a higher attention, more digits reflects a better short-term memory and a decreased latency indicates a increased visual information processing speed.

### Green space exposure

Residential addresses of the participants at the time of the cognitive examination were geocoded by using the Central Reference Address Database (CRAB) from the Agency for Geographic Information Flanders (AGIV). For those children who had more than one residential address at the time of the study, we calculated a weighted average refelecting the time spent at each location. We estimated the surface area of green space (%) and agricultural area (%) in several radii (50, 100, 300, 500, 1000, and 2000 m) around the residential address using the aerial-photo-based high-resolution (1 m^2^) land-cover dataset, Groenkaart Vlaanderen 2013 (Green Map of Flanders). The Green Map is established on raster-segmentation classification of the summer flight ortho-photos from 2012 [Agency for Nature and Forest (ANB) and AGIV] and provides high-resolution information on natural elements, identified as all non-agricultural vegetation and further refered to as green space. More information is provided by Dockx et al. 2022 [14]. All analyses were carried out using Geographic Information System (ArcGIS 10 software) functions.

### Assessment of air pollution and noise exposure

Daily residential exposure to particulate matter with aerodynamic diameter less than 2.5 µm (PM_2.5_) and NO_2_ (units: μg/m^3^) was estimated using a spatial–temporal interpolation method, which integrates the land-cover data obtained from satellite images (CORINE land-cover data set) [34] and air pollution data of fixed monitoring stations in combination with a dispersion model [35]. The dispersion model uses the results from the interpolation method as background and superimposes the effect of industrial point sources and line sources from traffic to calculate the daily concentration at high resolution. Model performance was evaluated by leave-one-out cross-validation and based on 34 monitoring points for PM_2.5_ and 44 for NO_2_. For Flanders, the interpolation tool gave a spatial–temporal explained variance of > 0.80 for PM_2.5_ and 0.78 for NO_2_ [36]. Chronic exposure was calculated by averaging the daily concentration over a one-year period that precedes the cognitive examination day. For children with more than one residential address at the moment of the study, we calculated a weighted average using the proportion of time spent at each location. Additionally, the residential distance to major roads, defined as highways and other national roads (AGIV), was calculated, using the ArcGIS 10 geographic information system functions.

Individual road noise exposure (dB), expressed as total exposure over an entire day (Lden), on the residential was calculated based on a region-wide noise map based on the environmental reporting (MIRA) from the Flanders Environment Agency (VMM.) The modelling of road noise level is provided for all main and secondary roads in Flanders and is calculated based on road traffic intensity and speed, vehicle-type-specific traffic density and type of street surface.

### Statistical analysis

We used SAS software, version 9.4 (SAS Institute, Cary, NC), for data management and statistical analyses. The associations between air pollutants and green space indicators were assessed using unadjusted Spearman’s correlations. Reported p-values were two-sided and were considered statistically significant when p < 0.05. In total 887 cognitive observations were available for analysis from the 307 children included in this study. To include all observations, we developed mixed effects models (unstructured covariance matrix) with study area (corresponding to the schools) and subject as random effects to assess the associations between green space and cognitive performance. In the main model, all covariates were a priori chosen, including sex (boy, girl), age (continuous, included ad linear – not transformed and quadratic term), BMI (continuous), maternal education (secondary education or lower, college/university degree), the highest rank of occupation of either parent (low, middle, high), passive smoking (yes, no), day of examination (continuous), and season of examination (spring, summer, autumn, winter) and neighbourhood household income (continuous). The non-linear effect of age was captured by the quadratic term. All covariates were recorded at baseline. The effect estimates were expressed for an interquartile range (IQR) increment of green space exposure within different radii from the residential address. The changes in outcomes for the different cognitive performance tests were characterized by different effect estimates: that is for the Continuous Performance Test and the Stroop Test, the change in msec for reaction time; for the Digit Span Forward and Backward Tests, the change in the number of digits; for the Digit-Symbol Test and Pattern Comparison Test, the change in sec for the latency.

We conducted a series of sensitivity analysis. First, we additionally adjusted the main model for ethnicity (both parents born in Belgium, one or both parents born abroad) and for time of examination (one at a time). The rationale to adjust for ethnicity is the possibility of ethnic inequalities in green space availability [37] or use [38]. Second, to test the association between agricultural area around the residence and the cognitive outcomes, we replaced green space with agricultural area in the main model. Third, we tested the effect modification of maternal education and sex. Maternal education, as indicator of socioeconomic status, might be a relevant modifier of the health benefits of green space exposure [36, 39]. If the interaction term (green space and maternal educational level) was significant (set at *p* ≤ 0.10), we stratified the analysis into two groups; children of mothers with a low educational level (*n* = 118) and high educational level (*n* = 189). Fourth, to check the effect modification by sex, we tested the interaction between green space and sex and stratified according to sex (154 boys, 153 girls).

The robustness of the findings was tested by further adjusting the main model for exposure to PM_2.5,_ NO_2_ during the year before examination, proximity to major roads or traffic noise (one at a time). To evaluate potential interaction effects of green space and air pollution, we specified interaction terms in the multi-exposure models. We assessed interaction effects by combining green space exposure with quintiles of air pollution exposure. Interaction effects were assessed on the multiplicative scale.

## Results

### Study population characteristics

The characteristics of the study participants together with their cognitive performance are summarized in Table 1 and the descriptive statistics for exposure variables are shown in Table 2. The children had a mean (± SD) age of 10.4 (± 1.2) years. The majority of the mothers (61.6%) had a college or university degree. The parents of most of the children (*n* = 158, 51.5%) were self-employed, specialist or had a management function. Exposure to passive smoking was reported for 41 (13.4%) participants. The median surface area (%) of green space within a 50–2000 m radius around the residence ranges from 52 to 56%. The median (IQR) concentration of exposure to PM_2.5_ was 14.9 µg/m^3^ (1.5) and to NO_2_ was 21.2 µg/m^3^ (2.6) and the median (IQR) residential distance to a major road was 330 m (648).

**Table 1:** Study population characteristics and cognitive performance outcomes of the participating schoolchildren (*n* = 307)

**Population characteristics**	
**Boys**	154 (50.2%)
**Age**	10.4 ± 1.2
**Body mass index, kg/m**^**2**^	17.2 ± 2.8
**Study area**	
Kiewit (Hasselt)	68 (22.1%)
Tienen	62 (20.2%)
Zonhoven	177 (57.7%)
**Education level of the mother**	
Secondary education or lower	118 (38.4%)
College or university degree	189 (61.6%)
**Highest parental occupational category of either parent**	
Unemployed or not qualified worker	26 (8.5%)
Qualified worker, white-collar assistant, or teaching staff	123 (40.0%)
Self-employed, specialist, or member of the management	158 (51.5%)
**Passive smoking**	41 (13.4%)
**Cognitive performance outcomes**	
**Sustained and selective attention**	
Continuous performance test, msec (*n* = 307)	593.3 ± 46.2
**Selective attention**	
Stroop test, msec (*n* = 305)	1424.0 ± 305.1
**Short-term memory**	
Digit span forward test, digits (*n* = 305)	5.2 ± 0.8
Digit span backward test, digits (*n* = 306)	4.0 ± 0.8
**Visual information processing speed**	
Pattern comparison test, sec (*n* = 299)	4.2 ± 0.9
Digit symbol test, sec (*n* = 307)	123.6 ± 21.5

Values represent number (%) or mean ± SD

**Table 2:** Environmental exposure characteristics at the residential address of the children (*n* = 307)

	**Mean (± SD)**	**Median**	**25**^**th**^** percentile**	**75**^**th**^** percentile**
**Surface area of green space within 6 buffers around the residence, %**				
50 m	49.1 ± 0.2	52	38	61
100 m	50.6 ± 0.2	53	40	61
300 m	51.2 ± 0.1	54	44	60
500 m	51.2 ± 0.1	53	44	61
1000 m	50.1 ± 0.1	53	49	57
2000 m	51.0 ± 0.2	56	48	59
**Air pollution and traffic-related exposure**				
PM_**2.5**_, µg/m^3^	14.9 ± 1.0	14.9	14.2	15.7
NO_**2**_, µg/m^3^	20.9 ± 2.0	21.2	19.6	22.2
Distance to major road, m	552 ± 560	330	127	775

For the sustained and selective attention, the reaction time of the Continuous Performance Test averaged 593.3 ± 46.2 ms; for selective attention, the reaction time of the Stroop Test averaged 1424.0 ± 305.1 ms; for short-term memory, the number of digits of the Digit Span Forward and Backward Tests averaged 5.2 ± 0.8 and 4.0 ± 0.8 digits respectively; and for the visual information processing speed, the latency of the Pattern Comparison and the Digit-Symbol Tests averaged 4.2 ± 0.9 s and 123.6 ± 21.5 s respectively.

### Spearman correlations between residential green space, air pollutants, and distance to major roads

Residential green space correlated weakly with NO_2_ levels (r_s_ = -0.23 to -0.39) and distance to major roads (r_s_ = 0.26 to 0.41), while inverse moderate to strong correlations were found with residential PM_2.5_ exposure (r_s_ = -0.42 to -0.66) (Supplemental Table 1A). The interrelationships between residential annual average exposure to NO_2_, PM_2.5_, and distance to major roads showed moderate to strong correlations (r_s_ = -0.53 for PM_2.5_—distance to major road, r_s_ = 0.61 for PM_2.5_—NO_2_, and r_s_ = -0.70 for NO_2_—distance to major road; Supplemental Table 1B).

### Association between green space exposure and cognitive performance

**Fig. 1:**
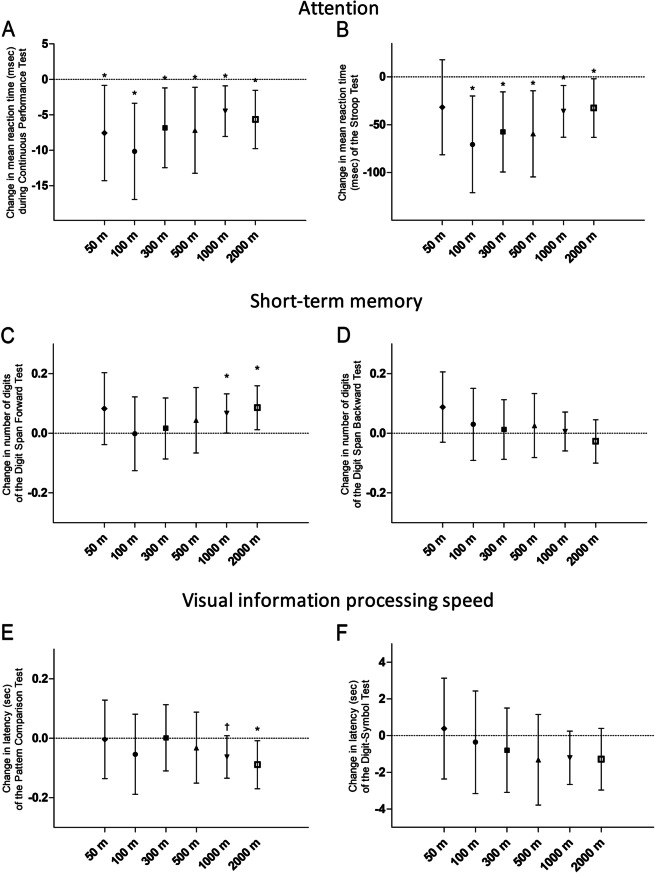
Association estimates between the change in the outcome of cognitive parameters and the IQR increment of percentage green space within several radii around the residence of the children. **A** Change in mean reaction time (msec) for sustained and selective attention in the Continuous Performance Test (*n* = 307); **B** Change in mean reaction time (msec) for selective attention in the Stroop Test (*n* = 305); **C** Change in the number of digits for the short-term memory in the Digit Span Forward Test (*n* = 305) and **D**. in the Digit Span Backward Test (*n* = 306); **E** Change in latency (sec) for the visual information processing speed in the Pattern Comparison Test (*n* = 299) and **F**. in the Digit-Symbol Test (*n* = 307). The main models (single exposure) were adjusted for sex, age (linear and quadratic term), BMI, education of the mother, highest occupation of either parent, passive smoking, day of the week, season of examination, neighbourhood household income, and the random effect of the study area and subject. Statistically significant estimates (*p* < 0.05) at a 95% confidence level are marked with an asterisk, and with † for borderline significance (0.05 < *p* < 0.10)

An IQR increment of green space exposure within 100–2000 m of the child’s residence was significantly associated with shorter reaction time in the sustained-selective (Continuous Performance Test) and selective (Stroop Test) attention test results after adjusting for sex, age (linear and quadratic term), BMI, education of the mother, highest occupation of either parent, passive smoking, day of the week, season of examination, and neighbourhood household income (Fig. 1, Supplement Table 2). For example, for an IQR increment of 21% in green space exposure in a 100 m buffer around the residence, the mean reaction time was 10.16 ms (95% CI: -17.0 to -3.4 ms, *p* = 0.004) lower for the Continuous Performance Test and 70.66 ms (95% CI: -121.2 to -20.1 ms, *p* = 0.006) lower for the Stroop Test. The adjusted estimated effects of each of the fixed covariates in the main model of green space and attention are presented in Supplement Table 3.

In addition to attention, more residential green space within a large radius (1000 and 2000 m) around the residence, was significantly associated with better short-term memory based on the Digit-Span Forward Test (0.07 digits, 95% CI: 0 to 0.13 digits, *p* = 0.05; and 0.09 digits, 95% CI: 0.01 to 0.16 digits, *p* = 0.02, respectively) but not on the Digit-Span Backward Test (Fig. 1C, D). For visual information processing speed (Pattern Comparison and Digit-Symbol Tests), a trend towards lower latency was observed with green space exposure within a 1000 m and 2000 m radius around the residence. The average latency of the Pattern Comparison Test was only significantly associated with residential green space within 2000 m (-0.09 s, 95% CI: -0.17 to -0.008 s, *p* = 0.03) (Fig. 1E). We observed no significant association between green space and latency of the Digit-Symbol Test.

In a sensitivity analysis, additional adjustment for ethnicity or time of examination did not result in a considerable change of the aforementioned effect estimates observed in our main analysis (Supplement Fig. 1 and 2). Opposite to green space, for surrounding agricultural area we noted a trend towards an inverse association with attention (Supplement Fig. 3). In general we did not observed an effect modification by maternal eduction on the association between residential green space and cognitive performance (Supplement Table 4). But in children of mothers with higher educational background, we noted a more pronounced associations between residential green space within 1000 m and 2000 m and the respectively attention outcomes based on the Continuous Performance Test (p-interaction green within 1000 m and education = 0.01; low education: 1.67 ms; 95% CI: -11.0 to 14.3; *p* = 0.80; high education: -7.08 ms; 95% CI: -11.3 to -2.3; *p* = 0.001) and on the Stroop Test (p-interaction green within 2000 m and education = 0.02; low education: -7.06 ms; 95% CI: -141.0 to 126.8; *p* = 0.92; high education: -32.67 ms; 95% CI: -50.4 to -15.0; *p* = 0.0004). In addition, stratified analysis showed more pronounced associations between residential green space within 300 m and 500 m and sustained attention among boys, p for interaction 0.09 (boys: -91.42 ms; 95% CI: -163.9 to -18.9; *p* = 0.01; girls: -34.01 ms; 95% CI: -84.3 to 16.2; *p* = 0.19) and 0.04 (boys: -101.63 ms; 95% CI: -177.6 to -25.7; *p* = 0.01; girls: -22.72 ms; 95% CI: -78.2 to 32.8; *p* = 0.42) respectively (Supplement Table 5).

**Fig. 2:**
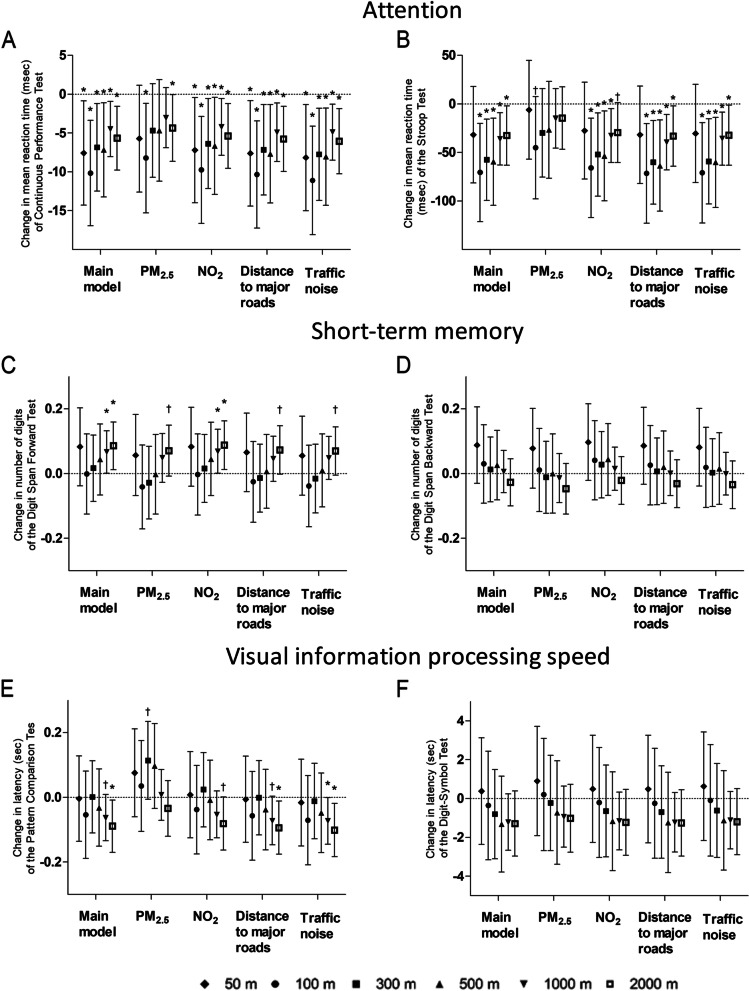
Comparison of the association estimates of the main models (single-exposure to green space) to those of the multi-exposure models as represented by the change in the outcome of cognitive parameters for the IQR increment in green space within several radii around the residence. **A** Change in mean reaction time (msec) for the Continuous Performance Test (*n* = 307); **B** Change in mean reaction time (msec) for the Stroop Test (*n* = 305); **C** Change in the number of digits for the Digit Span Forward Test (*n* = 305) and **D**. for the Digit Span Backward Test (*n* = 306); **E** Change in latency (sec) for the Pattern Comparison Test (*n* = 299) and **F**. for the Digit-Symbol Test (*n* = 307). The main models were adjusted for sex, age (linear and quadratic term), BMI, education of the mother, highest occupation of either parent, passive smoking, day of the week, season of examination, neighbourhood household income, and the random effect of the study area and subject. The multi-exposure models were additionally adjusted for exposure to PM_2.5_, NO_2_ during the year before examination, distance to major roads, or traffic noise. Statistically significant estimates (*p* < 0.05) at a 95% confidence level are marked with an asterisk, and with † for borderline significance (0.05 < *p* < 0.10)

### Cognition in multi-exposure models involving green space and traffic-related exposure

The associations between an IQR increment of green space within a 100 m – 2000 m radius around the residence and a better attention outcome as observed for the single-exposure models remained significant after additional adjustment for traffic-related air pollution exposure (NO_2_ exposure one year before examination), distance from the residence to the nearest major roads, or traffic noise (Fig. 2A, B). For example, significantly lower mean reaction times were observed for a 21% IQR increment in green space exposure within 100 m of the residence for both the sustained-selective attention outcome (-9.74 ms, 95% CI: -16.6 to -2.9 ms, *p* = 0.006) and the selective attention outcome (-65.90 ms, 95% CI: -117.0 to -14.8 ms, *p* = 0.01), and that independent of the annual average NO_2_ exposure at residence. Similarly, independent of the distance to major roads, significantly lower mean reaction times were observed for both the sustained-selective (-10.33 ms, 95% CI: -17.2 to -3.4 ms, *p* = 0.004) and selective attention outcomes (-71.63 ms, 95% CI: -122.9 to -20.4 ms, *p* = 0.006) for a 21% IQR increment of green space exposure within 100 m of the residence (Fig. 2A, B).

Similarly, short-term memory (Digit Span Forward Test) also remained significantly associated with green space (1000 m and 2000 m) after adjusting the main model for NO_2_ exposure, and borderline significant with green space (2000 m) after adjustment for distance to major roads (*p* = 0.057) or traffic noise (*p* = 0.066; Fig. 2C). For example, an IQR increment in residential green space (2000 m) persisted associated with a better short-term memory (0.09 digits, 95% CI: 0.01 to 0.16 digits, *p* = 0.023) on the Digit-Span Forward Test after additional adjustment for NO_2_. About the visual information processing speed (Pattern Comparison Test, Fig. 2E), residential green space within 2000 m remained significant after additional adjustment for NO_2_ exposure, distance to major roads or traffic noise associated with lower latency, borderline for NO_2_ (-0.08 s, 95% CI: -0.16 to 0.001 s, *p* = 0.054) and significantly for distance to major roads (-0.09 s, 95% CI: -0.18 to -0.01 s, *p* = 0.03) and for traffic noise (-0.10 s, 95% CI: -0.18 to -0.02 s, *p* = 0.02).

### Cognition in multi-exposure models involving green space and particulate air pollution

In multi-exposure models involving surrounding green space and adjustment for PM_2.5_ air pollution, the associations with the attention outcomes were attenuated (Fig. 2A, B), especially for the selective attention outcome. Only green space within a radius of 100 m and 2000 m of the residence remained significantly associated with sustained and selective attention independent of the PM_2.5_ exposure at residence. Hence, for an IQR increment (21% and 11%) of green space within 100 m and 2000 m of the residence, the sustained attention outcome showed a significantly lower mean reaction time (-8.23 ms, 95% CI: -15.3 to -1.2 ms, *p* = 0.02; and -4.35 ms, 95% CI: -8.6 to -0.07 ms, *p* = 0.05) (Fig. 2A). For selective attention, short-term memory, and visual information processing speed no significant associations were observed with green space after adding PM_2.5_ to the main models (Fig. 2B-F).

We evaluated the interaction between green space and particulate air pollution and hypothesized that the association between green space and cognition is the strongest in the lowest quintile of air pollution [40]. We found no significant interaction between green space and quintiles of air pollution on the attention outcomes. In the lowest quintile of PM_2.5_ exposure, we observed a significant association between green space close to the residence and both attention outcomes (Supplement Fig. 4). An IQR increase in residential green space within 100 m was associated with a relative strong decrease in reaction time on the continuous performance test and on the stroop test. This was not observed within third quintile and higher of PM_2.5_ exposure.

## Discussion

We evaluated the association of the exposure to green space with cognitive performance in a sample of primary schoolchildren in Belgium. We observed that green space exposure was associated with better cognitive performance in primary schoolchildren, which appeared to be independent of exposure to traffic-related air pollution exposure. Specifically, more green space exposure was associated with a significant improvement of sustained and selective attention. In addition, more green space exposure within a large radius (1000 to 2000 m) around the residence is significantly associated with a better outcome of short-term memory and visual information processing speed taking into account traffic-related exposure. In general, the association between green space and cognition did not differ by sex. Apart from sustained attention (based on the Stoop Test) which is related to green space within 300 m and 500 m among boys but not girls. There is no consensus in the literature but others found also higher risk for inattention with increasing distances to green only among males [41].

Our prospective study had a number of strengths. First, cognitive performance was assessed based on repeated measurements and covered four domains of cognition. A major strength of this study was that the percentage of green space around the residence was calculated based on a high resolution 1 × 1 m land cover map. Another advantage of the green space data used in this study is that it does not include agriculture green. In addition, residential air pollution exposure (NO_2_ and PM_2.5_) was estimated using a high-resolution spatial–temporal model. We acknowledge a few limitations to our study. A first limitation is that no data is available on room temperature. Besides the modest sample size, a limitation is the lack of information of time-activity patterns, such as time spend outdoors, and access to green space.

Previous results of the COGNAC study indicated that chronic PM_2.5_ air pollution exposure at the residence was adversely associated with the selective attention and sustained attention outcomes assessed with the Stroop and Continuous Performance Test [29]. Therefore, when investigating green space in association with cognitive health, it is important to take into account the potential interaction with outdoor air pollution. Previous research treated air pollution as a confounder or as a mediator based on the assumption of causality [23, 40]. Mediation analysis is warranted if green vegetation notabely reducing air pollution concentrations (e.g. through dry deposition), rather than green spaces being areas where air pollution sources are simply absent. In the latter case, air pollution could be considered as a confounder in models investigating green space and health outcomes. Our findings indicated that after additional adjustment for traffic-related air pollution exposure at the residence (NO_2_ and distance to major roads), the significant association remained between more residential green space and better attention outcome. In contrast, adjustment for PM_2.5_ air pollution, a pollutant with lower impact of local traffic, attenuated the associations between green space and cognitive outcomes. This is probably because surrounding green space correlated weakly with NO_2_, while with PM_2.5_ the correlations were moderately strong. A study based on personal levels of air pollution exposures also observed an association between residential surrounding green space and PM_2.5_ but there was no association with NO_2_ exposure [42]. Another complicating factor is that ambient air pollution is based on models including CORINE land-cover data. CORINE land-cover data does not capture small patches of green space since it only includes green areas that are at least 25 ha, whearas the green space used in this study is based on high-resolution (1 m^2^) data. Nevertheless, modelled air pollution cannot be considered completely independent from residential green space and, consequently, these results should be interpreted with caution.

Our finding of a positive association between green space exposure and attentiveness in children is consistent with the existing literature. An investigation based on 1500 children from the INMA cohort in Sabadell and Valencia (Spain) showed that more greenness around the residence of the children was associated with better scores on attention tests at the age of 4–5 and the age of 7 [43]. Among 456 children aged 4 to 6 years of the ENVIRONAGE birth, a beneficial influence of residential green space in close proximity (50–100 m) was observed on the attention and psychomotor speed, represented by the Motor Screening Task [14]. A longitudinal study in Italy found that residential surrounding greenness was associated with better scores on an attention test at 7 years of age, partly mediated by NO_2_ reduction [17]. The mediation analysis indicated that NO_2_ explained 35% (90% CI: 7%-62%) of the total effect of residential greenness within a 500 m buffer on the attention test outcome. In addition, another Spanish study exploring the mediating role of air pollution showed that the association between greenness and cognitive development could be partly mediated by reductions in air pollution [16]. They reported that indoor levels (at school) of elemental carbon explained 20–65% of the associations between green spaces and 12-month cognitive development [16].

In extent of our findings in children, green space in a 2000 m radius surrounding the residence and school combined was associated with better sustained and selected among Flemish adolescents between 13 and 17 years old [44]. The effect size observed in our study is comparable to results observed in adolescents as an IQR (13%) contrast in green space (residence-school) within 2000 m is associated with a 7.28 ms (95% CI:—11.7 to—2.8; *p* = 0.001) and a 32.7 ms (95% CI:—58.9 to—6.5; *p* = 0.02) decrease in reaction time based on the Continuous Performance Test and the Stroop Test, respectively [44]. A nationwide cohort study in the US of adult women, found that higher levels of residential green space were associated with higher scores on processing speed and attention and on overall cognition [45]. However, residential greenness was not associated with cognition (reaction time, working memory, and executive function) in adults residing in Quebec [46].

We observed mixed results for the short-term memory test since residential green space was found associated with better performance on the first part of the test (Digit Span Forward) requiring moderate attention, whereas no effect was found with the second part of the test requiring greater attention resources (Digit Span Backward). Similar to our findings, an experimental study in 28 persons showed that exercise in a natural environment resulted in the greatest improvement for cognitive tasks that required only moderate attentional demands [47]. A systematic review with a meta-analysis reported that the natural exposure group performed significantly better than controls on both Digit Span Forward and Digit Span Backward Tests [48]. However, other studies found no performance improvement on the Digit Span Test after walking in nature compared to walking in urban environments [49, 50]. The visual information processing speed outcome (Pattern Comparison and Digit-Symbol Tests) showed a trend of lower latency with more green space in a large radius around the residence. In contrast with our results on the Digit-Symbol Test, a meta-analysis based on two studies in adults reported the absence of association between exposure to nature and performance on a comparable test (Symbol Digit Modalities Test) [48]. To our knowledge, till now only one study investigated the association between green space or nature exposure and cognitive outcomes based on the Pattern Comparison Test. Contrary to our results in children, residential green space was not significantly associated with short-term memory and visual information processing speed among adolescents [44]. In the current study, better performance as to short-term memory and visuals information processing speed were only observed in association with green space in a large radius around the residence (1000 and 2000 m). Although we did not observe a substantial difference in the average percentage of green space within the various radii (50–2000 m), we assume that large connected green areas situated in a wide radius can differ in use which possibly explains the results.

Possible factors, besided air pollution exposure, that may explain the long-term benefits of green spaces are reduction in stress, an increase of physical activity, increased social contacts, mitigation of noise and heat [51]. Within environments with more green space, residentents are less exposed to noise [52] and heat [53]. This may reduce the deplation of cognitive resources [54, 55]. Besides environmental benefits, urban green space also promotes physical activity [56], social contacts [57, 58], stress reduction, and recovery from attention fatigue [59]. According to the attention restoration theory, interacting with nature grabs the involuntary attention and replenishes the voluntary attention that is directed by the cognitive-control process [60]. Experimental studies have shown that interacting with natural green environments could improve the performance on cognitive tasks that depend on directed-attention abilities [61]. The biodiversity hypothesis postulates that a healthy development of the human microbiota depends in parts on the inoculation with microbes from environmental sources [62]. In this way, environmental microbiota is a hypothesized mechanism involved in the relationship between green spaces and health [63]. Studies support this hypothesis by showing that the presence of green space determines the diversity of indoor environment [64] and human microbiota [65]. Microbial input from the environment may drive brain regulation and is a possible major component of the beneficial effect of greenness [66]. However, the exact mechanism of how green space exposure exerts its health benefits in children needs to be further elucidated as the available evidence is still limited.

## Conclusion

Our panel study showed that exposure to residential surrounding green space was associated with better sustained and selective attention performances in primary schoolchildren and that independent of traffic-related air pollution exposure. More green space within a large radius around the residence was associated with better performance as to short-term memory and visuals information processing speed. Further research is need to replicate our results in other settings and to investigate other critical windows of susceptibility to environmental exposures such as the prenatal period. Our findings in children strongly support the necessity of more green spaces in the residential environment to promote healthy cognitive development in childhood. Building and promoting attractive green for children may have lasting beneficial effects throughout their life course.

## Supplementary Information

**Additional file1: Supplement Figure 1. **Main model additionally adjusted for ethnicity. **Supplement Figure 2. **Main model additionally adjusted for time of examination. **Supplement Figure 3.** Association estimates between the change in the outcome of cognitive parameters and the IQR increment of percentage agricultural area within several radii around the residence of the children. **Supplement Figure 4.** Estimated change in cognitive parameters for an IQR increment of percentage green in quintiles of PM_2.5_air pollution exposure. **Supplement Table 1A.** Unadjusted Spearman’s correlation coefficients for the relationships between different green space buffers and air pollutants or distance to major roads (*n*=307). **Supplement Table 1B.** Unadjusted Spearman’s correlation coefficients for the intercorrelations between PM_2.5_, NO_2_, and distance to major roads (*n*=307). **Supplement Table 2**. Association estimates between the change in the outcome of cognitive parameters and the IQR increment of percentage green space within several radii around the residence of the children. **Supplement Table 3.** Adjusted estimated effect of each of the fixed covariates in the main model of green space within a 100 m radius around the residence and the outcome of attention. **Supplement Table 4.** Estimated change in cognitive parameters for an IQR increment of green space within several radii around the residence of children of mothers with a low (*n*=118) and high education (*n*=189).**Supplement Table 5.** Estimated change in cognitive parameters for an IQR increment of green space within several radii around the residence of boys (*n*=154) and girls (*n*=153).

## Data Availability

The datasets used and/or analysed during the current study are available from the corresponding author on reasonable request.

## References

[1] Dadvand P, Pujol J, Macia D, Martinez-Vilavella G, Blanco-Hinojo L, Mortamais M (2018). The association between lifelong greenspace exposure and 3-dimensional brain magnetic resonance imaging in Barcelona Schoolchildren. Environ Health Perspect.

[2] Engemann K, Pedersen CB, Arge L, Tsirogiannis C, Mortensen PB, Svenning JC (2019). Residential green space in childhood is associated with lower risk of psychiatric disorders from adolescence into adulthood. Proc Natl Acad Sci U S A.

[3] Islam MZ, Johnston J, Sly PD (2020). Green space and early childhood development: a systematic review. Rev Environ Health.

[4] de Keijzer C, Gascon M, Nieuwenhuijsen MJ, Dadvand P (2016). Long-term green space exposure and cognition across the life course: a systematic review. Curr Envrion Health Rep.

[5] McCormick R (2017). Does access to green space impact the mental well-being of children: a systematic review. J Pediatr Nurs.

[6] Jimenez MP, DeVille NV, Elliott EG, Schiff JE, Wilt GE, Hart JE (2021). Associations between nature exposure and health: a review of the evidence. Int J Environ Res Public Health.

[7] de Keijzer C, Bauwelinck M, Dadvand P (2020). Long-term exposure to residential greenspace and healthy ageing: a systematic review. Curr Environ Health Rep.

[8] Gascon M, Triguero-Mas M, Martínez D, Dadvand P, Rojas-Rueda D, Plasència A (2016). Residential green spaces and mortality: a systematic review. Environ Int.

[9] Cohen-Cline H, Turkheimer E, Duncan GE (2015). Access to green space, physical activity and mental health: a twin study. J Epidemiol Community Health.

[10] Beyer KMM, Kaltenbach A, Szabo A, Bogar S, Nieto FJ, Malecki KM (2014). Exposure to neighborhood green space and mental health: evidence from the survey of the health of Wisconsin. Int J Environ Res Public Health.

[11] Van Aart CJC, Michels N, Sioen I, De Decker A, Bijnens EM, Janssen BG (2018). Residential landscape as a predictor of psychosocial stress in the life course from childhood to adolescence. Environ Int.

[12] Browning M, Rigolon A (2019). School green space and its impact on academic performance: a systematic literature review. Int J Environ Res Public Health.

[13] Flouri E, Papachristou E, Midouhas E. The role of neighbourhood greenspace in children’s spatial working memory. Br J Educ Psychol. 2019;89(2):359–73. 10.1111/bjep.12243 PMC6563484 30187470

[14] Dockx Y, Bijnens EM, Luyten L, Peusens M, Provost E, Rasking L (2022). Early life exposure to residential green space impacts cognitive functioning in children aged 4 to 6 years. Environ Int.

[15] Dadvand P, Tischer C, Estarlich M, Llop S, Dalmau-Bueno A, Lopez-Vicente M (2017). Lifelong residential exposure to green space and attention: a population-based prospective study. Environ Health Perspect.

[16] Dadvand P, Nieuwenhuijsen MJ, Esnaola M, Forns J, Basagana X, Alvarez-Pedrerol M (2015). Green spaces and cognitive development in primary schoolchildren. Proc Natl Acad Sci U S A.

[17] Asta F, Michelozzi P, Cesaroni G, De Sario M, Davoli M, Porta D (2021). Green spaces and cognitive development at age 7 years in a Rome birth cohort: the mediating role of nitrogen dioxide. Environ Res.

[18] Julvez J, López-Vicente M, Warembourg C, Maitre L, Philippat C, Gützkow KB (2021). Early life multiple exposures and child cognitive function: a multi-centric birth cohort study in six European countries. Environ Pollut.

[19] Bijnens E, Derom C, Thiery E, Weyers S, Nawrot T (2020). Residential green space and child intelligence and behavior across urban, suburban, and rural areas in Belgium: a longitudinal birth cohort study of twins. PLoS Med.

[20] Lee KS, Kim BN, Cho J, Jang YY, Choi YJ, Lee WS (2021). Associations between surrounding residential greenness and intelligence quotient in 6-year-old children. Sci Total Environ.

[21] Luque-García L, Corrales A, Lertxundi A, Díaz S, Ibarluzea J. Does exposure to greenness improve children’s neuropsychological development and mental health? A Navigation Guide systematic review of observational evidence for associations. Environ Res. 2022;206:112599. 10.1016/j.envres.2021.112599 34932982

[22] Buczyłowska D, Zhao T, Singh N, Jurczak A, Siry A, Markevych I (2023). Exposure to greenspace and bluespace and cognitive functioning in children - A systematic review. Environ Res.

[23] Markevych I, Schoierer J, Hartig T, Chudnovsky A, Hystad P, Dzhambov AM (2017). Exploring pathways linking greenspace to health: Theoretical and methodological guidance. Environ Res.

[24] Calderón-Garcidueñas L, Mora-Tiscareño A, Styner M, Gómez-Garza G, Zhu H, Torres-Jardón R (2012). White matter hyperintensities, systemic inflammation, brain growth, and cognitive functions in children exposed to air pollution. J Alzheimers Dis.

[25] Chiu YH, Bellinger DC, Coull BA, Anderson S, Barber R, Wright RO (2013). Associations between traffic-related black carbon exposure and attention in a prospective birth cohort of urban children. Environ Health Perspect.

[26] Sunyer J, Esnaola M, Alvarez-Pedrerol M, Forns J, Rivas I, Lopez-Vicente M (2015). Association between traffic-related air pollution in schools and cognitive development in primary school children: a prospective cohort study. PLoS Med.

[27] AQEG (2018). Impacts of vegetation on urban air pollution (Air Quality Expert Group).

[28] Vanaken G-J, Danckaerts M. Impact of green space exposure on children’s and adolescents’ mental health: a systematic review. Int J Environ Res Public Health. 2018;15(12):2668. 10.3390/ijerph15122668 PMC6313536 30486416

[29] Saenen ND, Provost EB, Viaene MK, Vanpoucke C, Lefebvre W, Vrijens K (2016). Recent versus chronic exposure to particulate matter air pollution in association with neurobehavioral performance in a panel study of primary schoolchildren. Environ Int.

[30] Xavier Educational Software Ltd (2011). Multi-function stroop test.

[31] Letz R (2000). NES3 User’s Manual.

[32] White RF, James KE, Vasterling JJ, Letz R, Marans K, Delaney R (2003). Neuropsychological screening for cognitive impairment using computer-assisted tasks. Assessment.

[33] Riccio CA, Reynolds CR, Lowe P, Moore JJ (2002). The continuous performance test: a window on the neural substrates for attention?. Arch Clin Neuropsychol.

[34] Janssen S, Dumont G, Fierens F, Mensink C (2008). Spatial interpolation of air pollution measurements using CORINE land cover data. Atmos Environ.

[35] Lefebvre W, Degrawe B, Beckx C, Vanhulsel M, Kochan B, Bellemans T (2013). Presentation and evaluation of an integrated model chain to respond to traffic- and health-related policy questions. Environ Model Softw.

[36] Rigolon A, Browning M, McAnirlin O, Yoon HV (2021). Green space and health equity: a systematic review on the potential of green space to reduce health disparities. Int J Environ Res Public Health.

[37] Ferguson M, Roberts HE, McEachan RRC, Dallimer M (2018). Contrasting distributions of urban green infrastructure across social and ethno-racial groups. Landsc Urban Plan.

[38] Robinson T, Robertson N, Curtis F, Darko N, Jones CR (2022). Examining psychosocial and economic barriers to green space access for racialised individuals and families: a narrative literature review of the evidence to date. Int J Environ Res Public Health.

[39] Kabisch N, Marselle MR, Stadler J, Korn H, Irvine KN, Bonn A (2019). The Influence of Socio-economic and Socio-demographic Factors in the Association Between Urban Green Space and Health. Biodiversity and Health in the Face of Climate Change.

[40] Klompmaker JO, Janssen NAH, Bloemsma LD, Gehring U, Wijga AH, van den Brink C (2019). Associations of combined exposures to surrounding green, air pollution, and road traffic noise with cardiometabolic diseases. Environ Health Perspect.

[41] Markevych I, Tiesler CM, Fuertes E, Romanos M, Dadvand P, Nieuwenhuijsen MJ (2014). Access to urban green spaces and behavioural problems in children: results from the GINIplus and LISAplus studies. Environ Int.

[42] Dadvand P, de Nazelle A, Triguero-Mas M, Schembari A, Cirach M, Amoly E (2012). Surrounding greenness and exposure to air pollution during pregnancy: an analysis of personal monitoring data. Environ Health Perspect.

[43] Dadvand P, Tischer C, Estarlich M, Llop S, Dalmau-Bueno A, López-Vicente M (2017). Lifelong residential exposure to green space and attention: a population-based prospective study. Environ Health Perspect.

[44] Bijnens EM, Vos S, Verheyen VV, Bruckers L, Covaci A, De Henauw S (2022). Higher surrounding green space is associated with better attention in Flemish adolescents. Environ Int.

[45] Jimenez MP, Elliott EG, DeVille NV, Laden F, Hart JE, Weuve J (2022). Residential green space and cognitive function in a large cohort of middle-aged women. JAMA Netw Open.

[46] Hystad P, Payette Y, Noisel N, Boileau C (2019). Green space associations with mental health and cognitive function: results from the Quebec CARTaGENE cohort. Environ Epidemiol.

[47] Trammell JP, Aguilar SC (2020). Natural is not always better: the varied effects of a natural environment and exercise on affect and cognition. Front Psychol.

[48] Ohly H, White MP, Wheeler BW, Bethel A, Ukoumunne OC, Nikolaou V (2016). Attention restoration theory: a systematic review of the attention restoration potential of exposure to natural environments. J Toxicol Environ Health B Crit Rev.

[49] de Brito JN, Pope ZC, Mitchell NR, Schneider IE, Larson JM, Horton TH (2019). Changes in psychological and cognitive outcomes after green versus suburban walking: a pilot crossover study. Int J Environ Res Public Health.

[50] Fuegen K, Breitenbecher K (2018). walking and being outdoors in nature increase positive affect and energy. Ecopsychology.

[51] Dadvand P, Gascon M, Markevych I, Marselle MR, Stadler J, Korn H, Irvine KN, Bonn A (2019). Green spaces and child health and development. Biodiversity and health in the face of climate change.

[52] Gidlöf-Gunnarsson AÖE (2017). Noise and well-being in urban residential environments: the potential role of perceived availability to nearby green areas. Landsc Urban Plan.

[53] Jiang Y, Huang J, Shi T, Wang H (2021). Interaction of urban rivers and green space morphology to mitigate the urban heat island effect: case-based comparative analysis. Int J Environ Res Public Health.

[54] Klatte M, Bergstrom K, Lachmann T (2013). Does noise affect learning? A short review on noise effects on cognitive performance in children. Front Psychol.

[55] Panno A, Carrus G, Lafortezza R, Mariani L, Sanesi G (2017). Nature-based solutions to promote human resilience and wellbeing in cities during increasingly hot summers. Environ Res.

[56] Kondo MC, Fluehr JM, McKeon T, Branas CC (2018). Urban green space and its impact on human health. Int J Environ Res Public Health.

[57] Maas J, van Dillen SME, Verheij RA, Groenewegen PP (2009). Social contacts as a possible mechanism behind the relation between green space and health. Health Place.

[58] Dadvand P, Hariri S, Abbasi B, Heshmat R, Qorbani M, Motlagh ME (2019). Use of green spaces, self-satisfaction and social contacts in adolescents: a population-based CASPIAN-V study. Environ Res.

[59] Lee AC, Jordan HC, Horsley J (2015). Value of urban green spaces in promoting healthy living and wellbeing: prospects for planning. Risk Manag Healthc Policy.

[60] Kaplan S (1995). The restorative benefits of nature: Toward an integrative framework. J Environ Psychol.

[61] Berman MG, Jonides J, Kaplan S (2008). The cognitive benefits of interacting with nature. Psychol Sci.

[62] Sandifer PA, Sutton-Grier AE, Ward BP (2015). Exploring connections among nature, biodiversity, ecosystem services, and human health and well-being: opportunities to enhance health and biodiversity conservation. Ecosyst Serv.

[63] Logan AC (2015). Dysbiotic drift: mental health, environmental grey space, and microbiota. J Physiol Anthropol.

[64] Dockx Y, Täubel M, Bijnens EM, Witters K, Valkonen M, Jayaprakash B (2021). Residential green space can shape the indoor microbial environment. Environ Res.

[65] Selway CA, Mills JG, Weinstein P, Skelly C, Yadav S, Lowe A (2020). Transfer of environmental microbes to the skin and respiratory tract of humans after urban green space exposure. Environ Int.

[66] Rook GA (2013). Regulation of the immune system by biodiversity from the natural environment: an ecosystem service essential to health. Proc Natl Acad Sci U S A.

