# Supplementary material for: Residential green space improves cognitive performances in primary schoolchildren independent of traffic-related air pollution exposure
Source: Environ Health. 2023 Mar 30;22:33. doi: 10.1186/s12940-023-00982-z (PMC10061992; doi:10.1186/s12940-023-00982-z)
Supplement: Supplementary file 1 — Additional file1: Supplement Figure 1. Main model additionally adjusted for ethnicity. Supplement Figure 2. Main model additionally adjusted for time of examination. Supplement Figure 3. Association estimates between the change in the outcome of cognitive parameters and the IQR increment of percentage agricultural area within several radii around the residence of the children. Supplement Figure 4. Estimated change in cognitive parameters for an IQR increment of percentage green in quintiles of PM2.5air pollution exposure. Supplement Table 1A. Unadjusted Spearman’s correlation coefficients for the relationships between different green space buffers and air pollutants or distance to major roads (n=307). Supplement Table 1B. Unadjusted Spearman’s correlation coefficients for the intercorrelations between PM2.5, NO2, and distance to major roads (n=307). Supplement Table 2. Association estimates between the change in the outcome of cognitive parameters and the IQR increment of percentage green space within several radii around the residence of the children. Supplement Table 3. Adjusted estimated effect of each of the fixed covariates in the main model of green space within a 100 m radius around the residence and the outcome of attention. Supplement Table 4. Estimated change in cognitive parameters for an IQR increment of green space within several radii around the residence of children of mothers with a low (n=118) and high education (n=189).Supplement Table 5. Estimated change in cognitive parameters for an IQR increment of green space within several radii around the residence of boys (n=154) and girls (n=153). [file 12940_2023_982_MOESM1_ESM.docx]

**Supplementary material**

# Residential green space improves cognitive performances in primary schoolchildren independent of traffic-related air pollution exposure

Nelly D. Saenen^1^, Tim S. Nawrot^1,2^, Pauline Hautekiet^1,3^, Congrong Wang^1^, Harry A. Roels^1,4^, Payam Dadvand^5,6,7^, Michelle Plusquin^1^, Esmée M. Bijnens^1,8,9*^

**Affiliations**:

^1^ Centre for Environmental Sciences, Hasselt University, Hasselt, Belgium.
^2^ Department of Public Health and Primary Care, Leuven University, Leuven, Belgium.
^3^ Risk and Health Impact Assessment, Sciensano (Belgian Institute of Health), Brussels, Belgium.
^4^ Louvain Centre for Toxicology and Applied Pharmacology, Université catholique de Louvain, Brussels, Belgium.
^5^ ISGlobal, Barcelona, Spain.

^6^ Pompeu Fabra University, Barcelona, Catalonia, Spain.
^7^ Ciber on Epidemiology and Public Health (CIBERESP), Madrid, Spain.
^8^ Department of Human Structure and Repair, Ghent University Hospital, Ghent, Belgium
^9^ Department of Environmental Sciences, Faculty of Science, Open University, Heerlen, The Netherlands


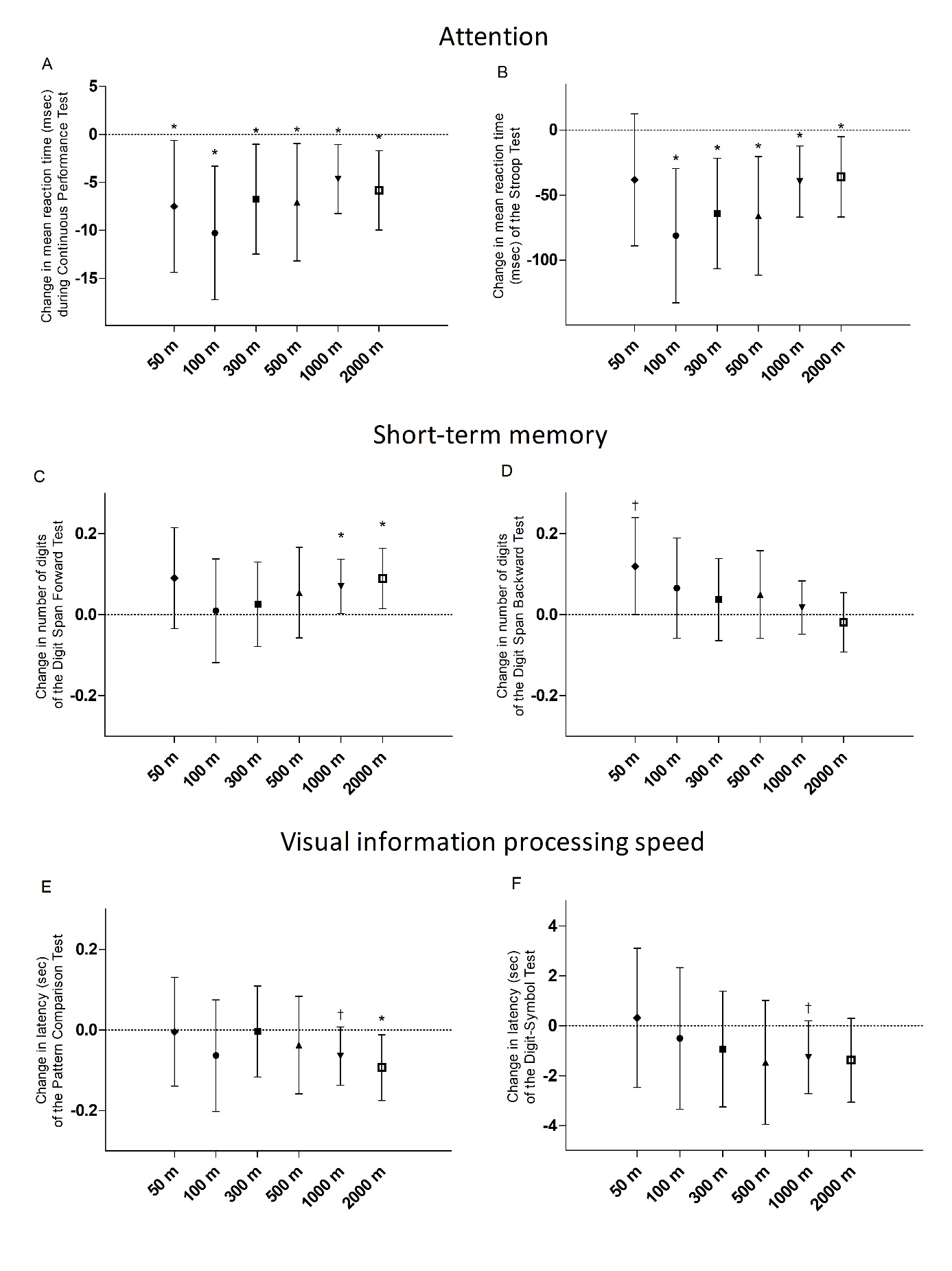
 **Supplement Figure 1. Main model additionally adjusted for ethnicity.** Estimated change in cognitive parameters for an IQR increment of percentage green space within several radii around the residence of the children. A. Change in mean reaction time (msec) for sustained attention in the Continuous Performance Test (n=307); B. Change in mean reaction time (msec) for selective attention in the Stroop Test (n=305); C. Change in the number of digits for the short-term memory in the Digit Span Forward Test (n=305) and D. in the Digit Span Backward Test (n=306); E. Change in latency (sec) for the visual information processing speed in the Pattern Comparison Test (n=299) and F. in the Digit-Symbol Test (n=307). The main models were adjusted for sex, age (linear and quadratic term), BMI, education of the mother, highest occupation of either parent, passive smoking, day of the week, season of examination, neighbourhood household income, and the random effect of the study area and subject. We additionally adjusted the main model for ethnicity. Statistically significant estimates (p < 0.05) at a 95% confidence level are marked with an asterisk, and with † for borderline significance (0.05 < p < 0.10).


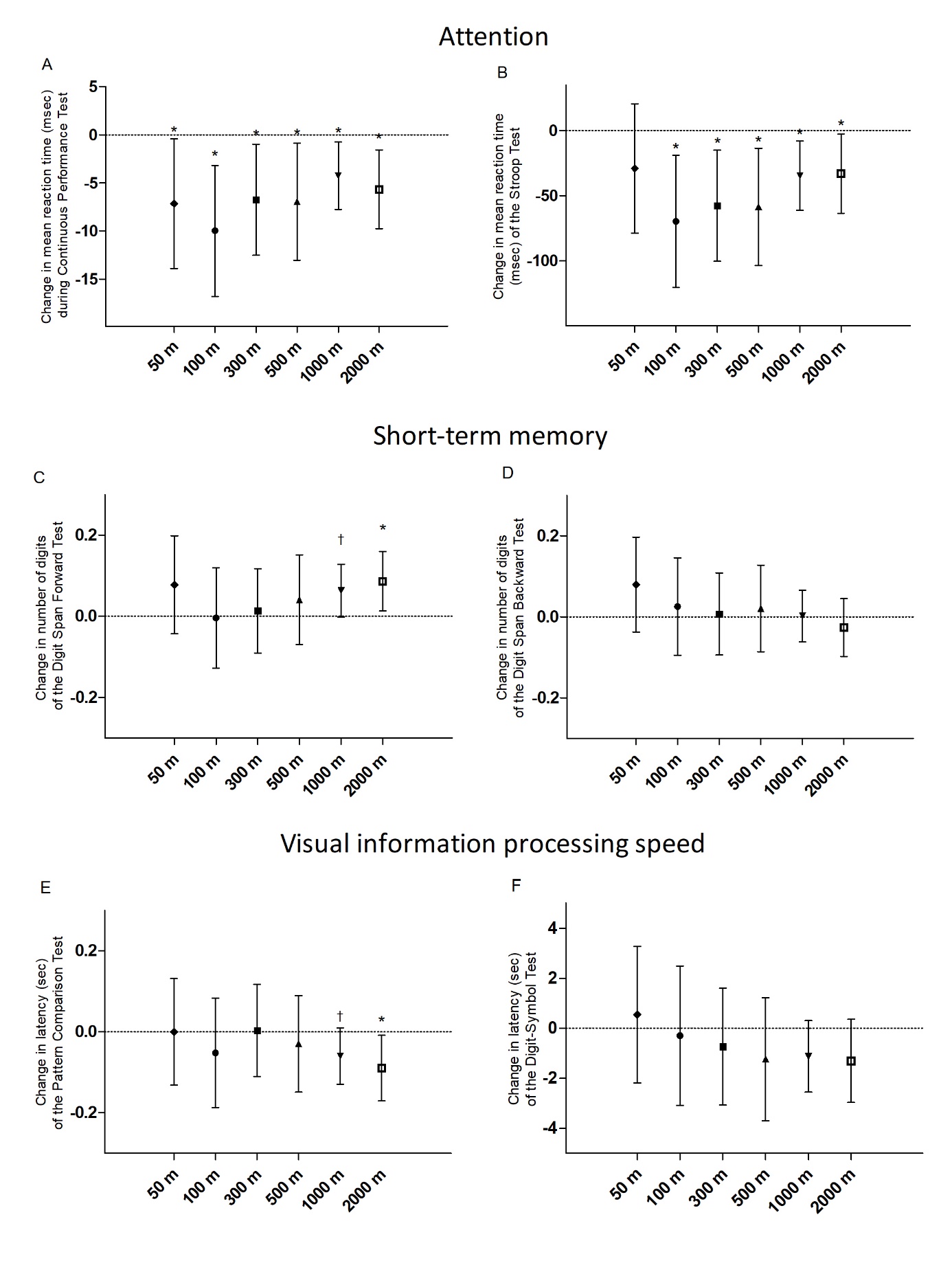


**Supplement Figure 2. Main model additionally adjusted for time of examination.** Estimated change in cognitive parameters for an IQR increment of percentage green space within several radii around the residence of the children. A. Change in mean reaction time (msec) for sustained and selective attention in the Continuous Performance Test (n=307); B. Change in mean reaction time (msec) for selective attention in the Stroop Test (n=305); C. Change in the number of digits for the short-term memory in the Digit Span Forward Test (n=305) and D. in the Digit Span Backward Test (n=306); E. Change in latency (sec) for the visual information processing speed in the Pattern Comparison Test (n=299) and F. in the Digit-Symbol Test (n=307). The main models (single exposure) were adjusted for sex, age (linear and quadratic term), BMI, education of the mother, highest occupation of either parent, passive smoking, day of the week, season of examination, neighbourhood household income, and the random effect of the study area and subject. We additionally adjusted the main model for examination time. Statistically significant estimates (p < 0.05) at a 95% confidence level are marked with an asterisk, and with † for borderline significance (0.05 < p < 0.10).


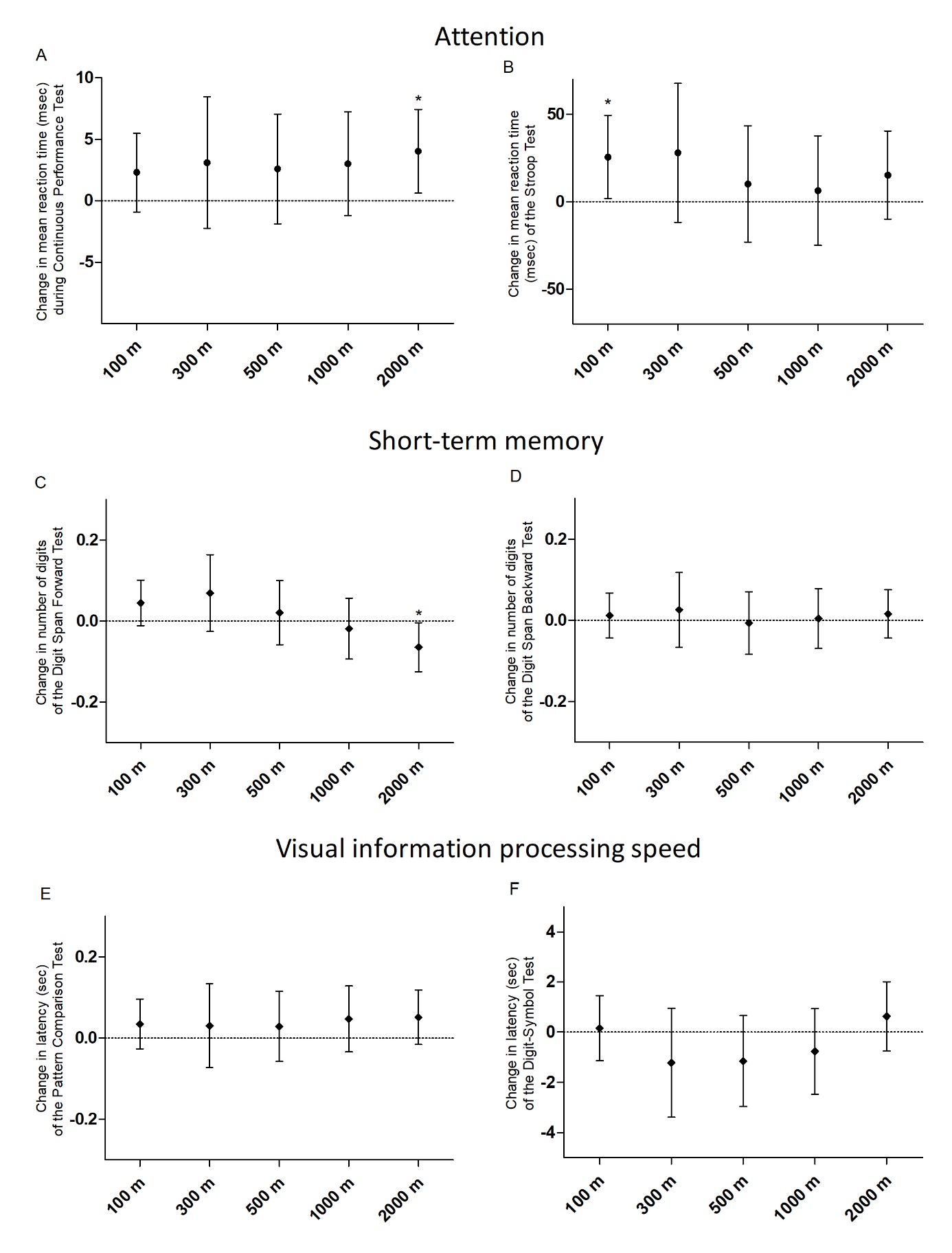
 **Supplement Figure 3. Association estimates between the change in the outcome of cognitive parameters and the IQR increment of percentage agricultural area within several radii around the residence of the children.** A. Change in mean reaction time (msec) for sustained and selective attention in the Continuous Performance Test (n=307); B. Change in mean reaction time (msec) for selective attention in the Stroop Test (n=305); C. Change in the number of digits for the short-term memory in the Digit Span Forward Test (n=305) and D. in the Digit Span Backward Test (n=306); E. Change in latency (sec) for the visual information processing speed in the Pattern Comparison Test (n=299) and F. in the Digit-Symbol Test (n=307). The models were adjusted for sex, age (linear and quadratic term), BMI, education of the mother, highest occupation of either parent, passive smoking, day of the week, season of examination, neighbourhood household income, and the random effect of the study area and subject.


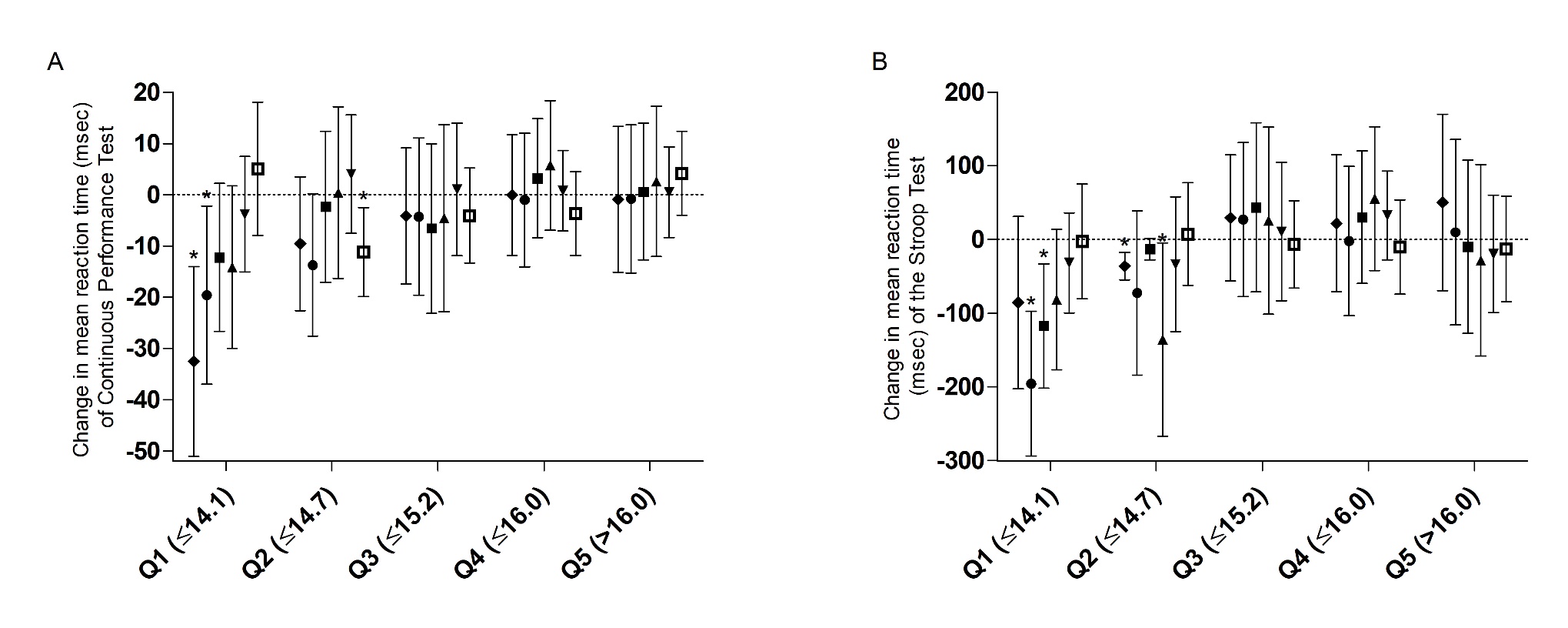
 **Supplement Figure 4. Estimated change in cognitive parameters for an IQR increment of percentage green in quintiles of PM_2.5_ air pollution exposure.** A. Change in mean reaction time (msec) for sustained attention in the Continuous Performance Test (n=307); B. Change in mean reaction time (msec) for selective attention in the Stroop Test (n=305) for an IQR increment of percentage green space within several radii around the residence of the children. The main models were adjusted for sex, age (linear and quadratic term), BMI, education of the mother, highest occupation of either parent, passive smoking, day of the week, season of examination, neighbourhood household income, and the random effect of the study area and subject. Statistically significant estimates (p < 0.05) at a 95% confidence level are marked with an asterisk.

| **Supplement Table 1A. Unadjusted Spearman’s correlation coefficients for the relationships between different green space buffers and air pollutants or distance to major roads (n=307).** | | | | | | |
| --- | --- | --- | --- | --- | --- | --- |
|  | **Green space within** | | | | | |
|  | **50 m** | **100 m** | **300 m** | **500 m** | **1000 m** | **2000 m** |
| **Chronic PM_2.5_** | -0.44 | -0.47 | -0.60 | -0.65 | -0.66 | -0.42 |
| **Chronic NO_2_** | -0.23 | -0.24 | -0.32 | -0.39 | -0.38 | -0.28 |
| **Distance to major roads** | 0.29 | 0.31 | 0.37 | 0.41 | 0.38 | 0.26 |

All correlation coefficients are p < 0.0001.

| **Supplement Table 1B. Unadjusted Spearman’s correlation coefficients for the intercorrelations between PM_2.5_, NO_2_, and distance to major roads (n=307).** | | | |  |
| --- | --- | --- | --- | --- |
|  | **Chronic PM_2.5_** | **Chronic NO_2_** | **Distance to major roads** | |
| **Chronic PM_2.5_** | 1 | 0.61 | -0.53 | |
| **Chronic NO_2_** | 0.61 | 1 | - 0.70 | |
| **Distance to major roads** | -0.53 | - 0.70 | 1 | |

All correlation coefficients are p < 0.0001.

| **Supplement Table 2.** Association estimates between the change in the outcome of cognitive parameters and the IQR increment of percentage green space within several radii around the residence of the children. | | | | | | | | | | | | | | | | | | | | | | | | |
| --- | --- | --- | --- | --- | --- | --- | --- | --- | --- | --- | --- | --- | --- | --- | --- | --- | --- | --- | --- | --- | --- | --- | --- | --- |
|  | **Attention** | | | | | | | |  | **Short-term memory** | | | | | | |  | **Visual information processing speed** | | | | | | |
|  | **Mean reaction time, msec (Continuous Performance Test)** | | | |  | **Mean reaction time, msec (Stroop Test)** | | |  | **Number of digits (Digit Span  Forward Test)** | | |  | **Number of digits (Digit Span Backward Test)** | | |  | **Latency, sec  (Pattern Comparison Test)** | | |  | **Latency, sec  (Digit-Symbol Test)** | | |
|  | **Esti-mate** | **Low 95% CI** | **High 95% CI** |  | | **Esti-mate** | **Low 95% CI** | **High 95% CI** |  | **Esti-mate** | **Low 95% CI** | **High 95% CI** |  | **Esti-mate** | **Low 95% CI** | **High 95% CI** |  | **Esti-mate** | **Low 95% CI** | **High 95% CI** |  | **Esti-mate** | **Low 95% CI** | **High 95% CI** |
| Green space |  |  |  |  | |  |  |  |  |  |  |  |  |  |  |  |  |  |  |  |  |  |  |  |
| 50 m | **-7.58** | **-14.3** | **-0.9** |  | | -31.78 | -81.4 | 17.9 |  | 0.08 | -0.04 | 0.20 |  | 0.09 | -0.03 | 0.21 |  | 0.00 | -0.14 | 0.13 |  | 0.38 | -2.4 | 3.1 |
| 100 m | **-10.16** | **-17.0** | **-3.4** |  | | **-70.66** | **-121.2** | **-20.1** |  | 0.00 | -0.13 | 0.12 |  | 0.03 | -0.09 | 0.15 |  | -0.05 | -0.19 | 0.08 |  | -0.36 | -3.2 | 2.4 |
| 300 m | **-6.84** | **-12.5** | **-1.2** |  | | **-57.55** | **-99.4** | **-15.7** |  | 0.02 | -0.09 | 0.12 |  | 0.01 | -0.09 | 0.11 |  | 0.00 | -0.11 | 0.11 |  | -0.80 | -3.1 | 1.5 |
| 500 m | **-7.18** | **-13.2** | **-1.1** |  | | **-59.58** | **-104.6** | **-14.6** |  | 0.04 | -0.07 | 0.15 |  | 0.03 | -0.08 | 0.13 |  | -0.03 | -0.15 | 0.09 |  | -1.32 | -3.8 | 1.2 |
| 1000 m | **-4.50** | **-8.1** | **-0.9** |  | | **-36.07** | **-63.1** | **-9.0** |  | **0.07** | **0.00** | **0.13** |  | 0.01 | -0.06 | 0.07 |  | -0.06 | -0.13 | 0.01 |  | -1.22 | -2.7 | 0.2 |
| 2000 m | **-5.66** | **-9.8** | **-1.5** |  | | **-****32.64** | **-63.2** | **-2.0** |  | **0.09** | **0.01** | **0.16** |  | -0.03 | -0.10 | 0.05 |  | **-0.09** | **-0.17** | **-0.01** |  | -1.29 | -3.0 | 0.4 |
|  |  |  |  |  | |  |  |  |  |  |  |  |  |  |  |  |  |  |  |  |  |  |  |  |
| The main models (single exposure) were adjusted for sex, age (linear and quadratic term), BMI, education of the mother, highest occupation of either parent, passive smoking, day of the week, season of examination, neighbourhood household income, and the random effect of the study area and subject. Statistically significant estimates (p < 0.05) at a 95% confidence level are in bold. | | | | | | | | | | | | | | | | | | | | | | | | |

| **Supplement Table 3.** Adjusted estimated effect of each of the fixed covariates in the main model of green space within a 100 m radius around the residence and the outcome of attention. | | | | | | | | | | | | |
| --- | --- | --- | --- | --- | --- | --- | --- | --- | --- | --- | --- | --- |
|  |  | **Mean reaction time, msec (Continuous Performance Test)** | | | | | **Mean reaction time, msec  (Stroop Test)** | | | | | |
|  | | | **Esti-mate** | **Low 95% CI** | **High 95% CI** | **P value** |  | | **Esti-mate** | **Low  95% CI** | **High 95% CI** | **P value** |
|  | | |  |  |  |  |  | |  |  |  |  |
| Boys | | | -7.3 | -16.3 | 1.8 | 0.12 |  | | -30.23 | -97.3 | 36.8 | 0.38 |
| Age, + 1 year | | | **-113.1** | **-177.1** | **-49.1** | **0.0006** |  | | **-908.0** | **-1404.4** | **-411.7** | **0.0004** |
| BMI | | | 0.7 | -5.0 | 6.5 | 0.80 |  | | 9.6 | -32.8 | 52.0 | 0.66 |
| Parental education level* | | | -2.8 | -13.2 | 7.6 | 0.60 |  | | 5.5 | -71.3 | 82.3 | 0.89 |
| Parental occupational* | | | -0.1 | -18.2 | 17.9 | 0.99 |  | | 91.8 | -41.1 | 224.7 | 0.18 |
| Not passive smoking | | | 0.8 | -13.2 | 14.8 | 0.91 |  | | 1.5 | -101.0 | 104.0 | 0.98 |
| Day of the week | | |  |  |  |  |  | |  |  |  |  |
| Monday vs Friday | | | 8.2 | -1.6 | 18.0 | 0.10 |  | | 73.2 | -10.4 | 156.8 | 0.09 |
| Tuesday vs Friday | | | -8.8 | -20.3 | 2.7 | 0.14 |  | | -21.8 | -112.5 | 68.9 | 0.64 |
| Wednesday vs Friday | | | 12.8 | -12.5 | 38.1 | 0.32 |  | | 52.6 | -143.0 | 248.2 | 0.60 |
| Thursday vs Friday | | | 1.0 | -9.6 | 11.6 | 0.86 |  | | **91.0** | **5.5** | **176.5** | **0.04** |
| Season of examination | | |  |  |  |  |  | |  |  |  |  |
| Spring vs winter | | | **-7.9** | **-14.1** | **-1.7** | **0.01** |  | | -23.6 | -83.6 | 36.4 | 0.44 |
| Summer vs winter | | | **14.8** | **10.4** | **19.1** | **<.0001** |  | | **206.1** | **162.4** | **249.8** | **<.0001** |
| Neighbourhood household income, +IQR | | | 2.3 | -4.7 | 9.4 | 0.52 |  | | 12.7 | -40.5 | 66.0 | 0.64 |
| Green space 100 m, +IQR | | | **-10.2** | **-17.0** | **-3.4** | **0.0036** |  | | **-70.7** | **-121.2** | **-20.1** | **0.0064** |
|  | | | | | | | | | | | | |
| CI confidence intervals, IQR interquartile range, *low vs high. Statistically significant estimates (p < 0.05) at a 95% confidence level are in bold. | | | | | | | | | | | | |

| **Supplement Table 4.** Estimated change in cognitive parameters for an IQR increment of green space within several radii around the residence of children of mothers with a low (n=118) and high education (n=189). | | | | | | | | | | | | | | | | | |  |
| --- | --- | --- | --- | --- | --- | --- | --- | --- | --- | --- | --- | --- | --- | --- | --- | --- | --- | --- |
|  |  | | **Low maternal education** | | | | | | | **High maternal education** | | | | | | | |  |
| Green space | p-interaction  green*edcuation | Esti- mate | | Low 95% CI | | High 95% CI | p-value | | | | Esti-mate | | Low 95% CI | | High 95% CI | | p-value | |
| **Mean reaction time, msec (Continuous Performance Test)** | | | | | | | | | | | | | | | | | |  |
| 50 m | 0.16 |  | |  | |  | | |  | |  | |  | |  | |  | |
| 100 m | 0.12 |  | |  | |  | | |  | |  | |  | |  | |  | |
| 300 m | 0.39 |  | |  | |  | | |  | |  | |  | |  | |  | |
| 500 m | 0.30 |  | |  | |  | | |  | |  | |  | |  | |  | |
| 1000 m | 0.01 * | 1.67 | | -10.95 | | 14.29 | | | 0.80 | | -7.08 | | -11.34 | | -2.82 | | 0.001 * | |
| 2000 m | 0.15 |  | |  | |  | | |  | |  | |  | |  | |  | |
| **Mean reaction time, msec (Stroop Test)** | | | | | | | | | | | | | |  | |  | |  |
| 50 m | 0.09 * | 9.23 | | -98.39 | | 116.85 | | | 0.87 | | -47.78 | | -95.94 | | 0.39 | | 0.05 | |
| 100 m | 0.54 |  | |  | |  | | |  | |  | |  | |  | |  | |
| 300 m | 0.83 |  | |  | |  | | |  | |  | |  | |  | |  | |
| 500 m | 0.45 |  | |  | |  | | |  | |  | |  | |  | |  | |
| 1000 m | 0.21 |  | |  | |  | | |  | |  | |  | |  | |  | |
| 2000 m | 0.02 * | -7.06 | | -140.96 | | 126.83 | | | 0.92 | | -32.67 | | -50.39 | | -14.96 | | 0.0004 * | |
| **Number of digits (Digit Span Forward Test)**  **Performance Test** | | | | | | | | | | | | | | | | | |  |
| 50 m | 0.74 |  | |  | |  | | |  | |  | |  | |  | |  | |
| 100 m | 0.53 |  | |  | |  | | |  | |  | |  | |  | |  | |
| 300 m | 0.99 |  | |  | |  | | |  | |  | |  | |  | |  | |
| 500 m | 0.88 |  | |  | |  | | |  | |  | |  | |  | |  | |
| 1000 m | 0.63 |  | |  | |  | | |  | |  | |  | |  | |  | |
| 2000 m | 0.56 |  | |  | |  | | |  | |  | |  | |  | |  | |
| **Number of digits (Digit Span Backward Test)**  **Performance Test** | | | | |  | | |  | |  | |  | |  | |  | |  |
| 50 m | 0.25 |  | |  | |  | | |  | |  | |  | |  | |  | |
| 100 m | 0.35 |  | |  | |  | | |  | |  | |  | |  | |  | |
| 300 m | 0.07 * | 0.13 | | -0.06 | | 0.32 | | | 0.19 | | -0.05 | | -0.18 | | 0.08 | | 0.42 | |
| 500 m | 0.13 |  | |  | |  | | |  | |  | |  | |  | |  | |
| 1000 m | 0.72 |  | |  | |  | | |  | |  | |  | |  | |  | |
| 2000 m | 0.89 |  | |  | |  | | |  | |  | |  | |  | |  | |
| **Latency, sec (Pattern Comparison Test)**  **Performance Test** | | | | | | | |  | |  | |  | |  | |  | |  |
| 50 m | 0.23 |  | |  | |  | | |  | |  | |  | |  | |  | |
| 100 m | 0.70 |  | |  | |  | | |  | |  | |  | |  | |  | |
| 300 m | 0.58 |  | |  | |  | | |  | |  | |  | |  | |  | |
| 500 m | 0.30 |  | |  | |  | | |  | |  | |  | |  | |  | |
| 1000 m | 0.14 |  | |  | |  | | |  | |  | |  | |  | |  | |
| 2000 m | 0.12 |  | |  | |  | | |  | |  | |  | |  | |  | |
| **Latency, sec (Digit-Symbol Test)** | | | | |  | | |  | |  | |  | |  | |  | |  |
| 50 m | 0.15 |  | |  | |  | | |  | |  | |  | |  | |  | |
| 100 m | 0.36 |  | |  | |  | | |  | |  | |  | |  | |  | |
| 300 m | 0.78 |  | |  | |  | | |  | |  | |  | |  | |  | |
| 500 m | 0.60 |  | |  | |  | | |  | |  | |  | |  | |  | |
| 1000 m | 0.18 |  | |  | |  | | |  | |  | |  | |  | |  | |
| 2000 m | 0.70 |  | |  | |  | | |  | |  | |  | |  | |  | |
| *The models were adjusted for the same covariates as the main model. Statistically significant estimates (p < 0.05) and interaction (level set at p < 0.10) are represented by an asterisk (*).* | | | | | | | | | | | | | | | | | |  |

| **Supplement Table 5.** Estimated change in cognitive parameters for an IQR increment of green space within several radii around the residence of boys (n=154) and girls (n=153). | | | | | | | | | | | | | |  |
| --- | --- | --- | --- | --- | --- | --- | --- | --- | --- | --- | --- | --- | --- | --- |
|  | |  | | **Boys** | | | | | | **Girls** | | | |  |
| Green space | p-interaction  green*sex | | Esti-mate | | Low 95% CI | High 95% CI | p-value | | Esti-mate | | Low 95% CI | High 95% CI | p-value | |
| **Mean reaction time, msec (Continuous Performance Test)** | | | | | | | | | | | | | | |
| 50 m | 0.70 | |  | |  |  | |  |  | |  |  |  | |
| 100 m | 0.61 | |  | |  |  | |  |  | |  |  |  | |
| 300 m | 0.22 | |  | |  |  | |  |  | |  |  |  | |
| 500 m | 0.29 | |  | |  |  | |  |  | |  |  |  | |
| 1000 m | 0.37 | |  | |  |  | |  |  | |  |  |  | |
| 2000 m | 0.52 | |  | |  |  | |  |  | |  |  |  | |
| **Mean reaction time, msec (Stroop Test)** | | | | | | | | | | | |  |  | |
| 50 m | 0.39 | |  | |  |  | |  |  | |  |  |  | |
| 100 m | 0.22 | |  | |  |  | |  |  | |  |  |  | |
| 300 m | 0.09 * | | -91.42 | | -163.89 | -18.96 | | 0.01 * | -34.01 | | -84.25 | 16.23 | 0.19 | |
| 500 m | 0.04 * | | -101.63 | | -177.61 | -25.65 | | 0.01 * | -22.72 | | -78.19 | 32.75 | 0.42 | |
| 1000 m | 0.13 | |  | |  |  | |  |  | |  |  |  | |
| 2000 m | 0.69 | |  | |  |  | |  |  | |  |  |  | |
| **Number of digits (Digit Span Forward Test)**  **Performance Test** | | | | | | | | | | | | | | |
| 50 m | 0.84 | |  | |  |  | |  |  | |  |  |  | |
| 100 m | 0.34 | |  | |  |  | |  |  | |  |  |  | |
| 300 m | 0.73 | |  | |  |  | |  |  | |  |  |  | |
| 500 m | 0.88 | |  | |  |  | |  |  | |  |  |  | |
| 1000 m | 0.60 | |  | |  |  | |  |  | |  |  |  | |
| 2000 m | 0.49 | |  | |  |  | |  |  | |  |  |  | |
| **Number of digits (Digit Span Backward Test)**  **Performance Test** | | | | | |  | |  |  | |  |  |  | |
| 50 m | 0.62 | |  | |  |  | |  |  | |  |  |  | |
| 100 m | 0.53 | |  | |  |  | |  |  | |  |  |  | |
| 300 m | 0.13 | |  | |  |  | |  |  | |  |  |  | |
| 500 m | 0.29 | |  | |  |  | |  |  | |  |  |  | |
| 1000 m | 0.82 | |  | |  |  | |  |  | |  |  |  | |
| 2000 m | 0.47 | |  | |  |  | |  |  | |  |  |  | |
| **Latency, sec (Pattern Comparison Test)**  **Performance Test** | | | | | | | |  |  | |  |  |  | |
| 50 m | 0.69 | |  | |  |  | |  |  | |  |  |  | |
| 100 m | 0.74 | |  | |  |  | |  |  | |  |  |  | |
| 300 m | 0.44 | |  | |  |  | |  |  | |  |  |  | |
| 500 m | 0.41 | |  | |  |  | |  |  | |  |  |  | |
| 1000 m | 0.69 | |  | |  |  | |  |  | |  |  |  | |
| 2000 m | 0.86 | |  | |  |  | |  |  | |  |  |  | |
| **Latency, sec (Digit-Symbol Test)** | | | | | |  | |  |  | |  |  |  | |
| 50 m | 0.86 | |  | |  |  | |  |  | |  |  |  | |
| 100 m | 0.62 | |  | |  |  | |  |  | |  |  |  | |
| 300 m | 0.43 | |  | |  |  | |  |  | |  |  |  | |
| 500 m | 0.2 | |  | |  |  | |  |  | |  |  |  | |
| 1000 m | 0.67 | |  | |  |  | |  |  | |  |  |  | |
| 2000 m | 0.71 | |  | |  |  | |  |  | |  |  |  | |
| *The models were adjusted for the same covariates as the main model. Statistically significant estimates (p < 0.05) and interaction (level set at p < 0.10) are represented by an asterisk (*).* | | | | | | | | | | | | | |  |
